# Parental Identity Development Processes as Predictors of Personal Growth During the Transition to Parenthood Among First-Time Expecting Mothers and Fathers

**DOI:** 10.3390/bs16050790

**Published:** 2026-05-15

**Authors:** Orit Eliakim, Neta Hikri-Boton, Nir Madjar, Elli Schachter, Maya Cohen-Malayev

**Affiliations:** 1 Faculty of Education, Bar Ilan University, Ramat Gan 529002, Israel; 2 School of Education, Tel Aviv University, Tel-Aviv 6997801, Israel

**Keywords:** personal growth, parental identity, transition to parenthood, developmental crisis

## Abstract

Objective: Using a two-point design, this study examined five parental identity development processes as predictors of personal growth following birth among first-time expecting mothers and fathers. Background: Although personal growth during the transition to parenthood has been documented and several factors promoting it have been identified, much remains unknown about what enables people to grow through this experience. Parental identity development, which has been linked to positive adjustment and well-being during this transition, represents an unexplored framework for understanding personal growth during this critical period. Method: A total of 169 first-time expectant parents (97 women, Mage = 31.9, SD = 3.46; 72 men, Mage = 32.1, SD = 3.43) participated in this two-point design study: 1–3 months before birth (T1) and 3–5 months after birth (T2). Results: Women showed an increase in personal growth following birth, while men showed a decrease. Only Identification with Commitment—one of the five parental identity processes—measured before birth predicted personal growth after birth for both genders. Among men, increases in three of these processes were associated with decreases in personal growth. Conclusions: These findings highlight the complex and gendered relationship between parental identity development and personal growth during the transition to parenthood, suggesting that these two frameworks are mutually informative: parental identity processes predict the emergence of personal growth, while personal growth outcomes reveal the complexity and nuance of parental identity formation during this pivotal period.

## 1. Introduction

“Well, I just heard the news today

It seems my life is going to change

I closed my eyes, begin to pray

Then tears of joy stream down my face”

(Stapp, 1999, “With Arms Wide Open”)

Parenthood is undoubtedly one of the most significant and transformative experiences in adult life, recognized as a defining moment in the human story (Cowan & Cowan, 2000; Feeney et al., 2001). For most people, being a parent is a profound source of meaning, purpose, and identity (Grundström et al., 2024; Katz-Wise et al., 2010)—one that not only enriches well-being but also fosters personal development and transformation (Porat-Zyman et al., 2017; Taubman-Ben-Ari et al., 2011). Indeed, research links parenthood to an increased sense of meaning in life, overall well-being, and positive psychological change (Grundström et al., 2024; Radó, 2020; Taubman-Ben-Ari et al., 2024). Among the most studied aspects of this experience is the critical period of the transition to parenthood—from pregnancy to a few months following birth (Adamsons, 2013; Genesoni & Tallandini, 2009). This period constitutes a significant turning point with profound implications for the lives of new fathers and mothers (Feeney et al., 2001; Cowan & Cowan, 1995).

Alongside its profound rewards, this transition is widely recognized as a normative developmental crisis characterized by a high concentration of intense challenges and pressures that appear simultaneously within a short period (Taubman-Ben-Ari et al., 2011). These challenges may lead to stress (Reid & Taylor, 2015), anxiety (Don et al., 2014), and postpartum depression (Liu et al., 2022; O’Hara & McCabe, 2013), difficulties that can have long-term effects (Keizer et al., 2010; Sasayama et al., 2025). Yet, these challenges can also serve as a catalyst for personal growth (PG)—positive psychological changes that result from struggling with significant life challenges (Taubman-Ben-Ari et al., 2011, 2019; Tedeschi et al., 2018).

Personal growth during the transition to parenthood has been documented among both mothers and fathers at various points during pregnancy and postpartum (Taubman-Ben-Ari et al., 2011, 2012, 2022; Porat-Zyman et al., 2017). However, the factors that promote personal growth during this transition remain insufficiently understood (Porat-Zyman et al., 2017). Understanding what enables people to grow through this developmental crisis is therefore a crucial theoretical and practical question—one that this study seeks to address by examining the role of parental identity development.

A significant factor linked to positive outcomes, such as improved adjustment and well-being among parents, is the development of parental identity (Fadjukoff et al., 2016; Meca et al., 2020; Piotrowski, 2018). This suggests that the processes through which parents construct their parental identity may also be associated with positive psychological outcomes such as personal growth. Despite the potential importance of parental identity development, studies on this topic remain relatively scarce (Piotrowski, 2018, 2021b). Knowledge of the underlying processes of parental identity development remains limited (Naudé & Piotrowski, 2022; Piotrowski, 2020). To better understand how parental identity development relates to personal growth, an important approach is to examine the internal processes of parental identity development throughout this key transitional period. This study joins several recent studies that have examined parental identity development through identity processual models (Meca et al., 2020; Piotrowski, 2018, 2020; Schrooyen et al., 2021), thereby addressing this knowledge gap.

This study was conducted among men and women expecting their first child, examining parental identity development and personal growth at two time points: 1–3 months before birth (T1) and 3–5 months after birth (T2). The aim was to examine whether and how parental identity processes during this key transitional period predict personal growth after birth—thus using the transition to parenthood as a unique window into the potential associations between identity development and positive psychological change. To our knowledge, the few studies that have examined parental identity development from a processual approach have examined it either among expectant or current parents (Eliakim et al., 2025; Meca et al., 2020) but have not followed the development of parental identity over time throughout the critical period of the transition to parenthood. Therefore, this is the first two-point design study that uses a processual model to examine the development of parental identity and its role in predicting personal growth during this pivotal life transition.

We begin by outlining the concept of parental identity and reviewing the progress made in this field, focusing on processual models that examine the underlying mechanisms and dynamics of parental identity development (Piotrowski, 2018). We then present the Five Dimensions of Identity Development Scale, adapted to Parental Identity (DIDS-PI; Eliakim et al., 2025), which serves as the processual framework used in this study. Next, we examine personal growth within the context of the transition to parenthood, exploring how the theoretical framework of parental identity development may serve as a lens for understanding what enables people to grow through this developmental crisis. Finally, we introduce the present study.

### 1.1. Parental Identity

The Eriksonian approach to identity examines the ways individuals answer the fundamental question “Who am I?” (Erikson, 1950), defining ego identity as the development of an inner feeling of both consistency and continuity over time (Erikson, 1968). Erikson (1968), in his eight stages of psychosocial development theory, claims that identity formation is the main developmental task of adolescence; however, in each subsequent stage, identity must be examined and reformulated, primarily in response to significant life events. This ongoing process becomes especially pronounced during major life transitions such as becoming a parent, one of the most life-changing events (Twenge et al., 2003). During this transition, former identity formation challenges are rekindled based on current developmental experiences, requiring individuals to establish a renewed identity that builds upon their existing identity (Katz-Wise et al., 2010).

Parental identity can be defined, across various theoretical models, as a person’s self-definition and mental representation of oneself as a parent (Delmore-Ko et al., 2000; Petit et al., 2017), the degree of importance of the parenting role (Maurer et al., 2001), its salience (Gaunt & Scott, 2014; Knoester & Petts, 2017), and the attitudes, emotions, and beliefs relating to the parental role (Raskin, 2002). These studies indicate the importance of parental identity for the mental health and well-being of the parent (Baldwin et al., 2018; Knoester & Petts, 2017) and the parent’s involvement in childcare (Gaunt & Scott, 2014). This, in turn, influences the development and well-being of the child (Delmore-Ko et al., 2000). In the present study, we examine parental identity from a neo-Eriksonian developmental psychological perspective, using a processual approach that views parental identity as a dynamic and evolving structure and focusing on the internal processes involved in its development. At the same time, we acknowledge that our perspective should be complemented by other approaches that focus, for example, on external factors—such as role theory (Turner, 2001) or on interactional–situational approaches—such as those suggested by Stryker’s identity theory (Serpe & Stryker, 2011).

### 1.2. Parental Identity Development

The development of parental identity is a dynamic process that often has roots in earlier periods of a person’s life, sometimes already in adolescence, through expectations and beliefs about life as a parent (Delmore-Ko et al., 2000; Gyberg & Frisén, 2017). This process is shaped by gendered socialization experiences in childhood (Endendijk & Portengen, 2022) as well and by internalized societal norms regarding traditional gender roles (Kaźmierczak & Karasiewicz, 2019). Parental identity continues to develop and is re-evaluated when a person is expecting their first child (Meca et al., 2020), after birth, and later on, in response to internal and external factors in the parent’s life (Fadjukoff et al., 2016; Piotrowski, 2018).

Recent multidimensional and processual approaches to parental identity (Gyberg & Frisén, 2017; Meca et al., 2020; Piotrowski, 2018; Schrooyen et al., 2021) are grounded in the neo-Eriksonian identity status paradigm of Marcia (1966), which identifies two core internal processes of identity development: exploration, an active process of examining and considering multiple alternative identities, and commitment, an active decision to adopt one or several identity alternatives. Within these models, parental identity is measured through the strength of commitment to the parenting role and the extent of exploration of parenting-related issues (Fadjukoff et al., 2016).

Processual parental identity studies overwhelmingly indicate the crucial role of parental identity development for the well-being of the parent (Fadjukoff et al., 2016; Meca et al., 2020; Piotrowski, 2018) and the importance of establishing a stable, coherent, and well-defined parental identity (Fadjukoff et al., 2016). For example, Meca et al. (2020), who examined parental identity among current and expectant parents, found that commitment has a negative correlation with anxiety and depressive symptoms and a positive correlation with different aspects of well-being and satisfaction. Additionally, Piotrowski (2021b) found that people who later declared regretting parenthood showed low commitment and low identification with commitment to the parental role and low motivation to go through exploration processes in the parental domain. Schrooyen et al. (2021) found that a well-defined parental identity served as a protective factor against negative mental health outcomes during the stressful period of COVID-19. The current study employs the Dimensions of Identity Development Scale to measure the internal processes of parental identity development during the transition to parenthood.

#### 1.2.1. The Dimensions of Identity Development Scale—Parental Identity (DIDS-PI)

The Dimensions of Identity Development Scale (DIDS; Luyckx et al., 2008) is based on a detailed and dynamic process-oriented model of identity development. This dual-cycle model includes a commitment formation cycle, which involves forming stable and strong commitments, and a commitment evaluation cycle, which involves evaluating current commitments. The model includes three distinct dimensions of exploration and two distinct dimensions of identity commitment, with each dimension describing a different identity development process (Luyckx et al., 2008). Originally developed to measure nonspecific future plans, the DIDS has since been adapted to various specific identity domains. The Dimensions of Identity Development Scale, adapted to Parental Identity (DIDS-PI; Eliakim et al., 2025), represents an adaptation developed and validated specifically for parental identity among current and expectant parents. The DIDS-PI (Eliakim et al., 2025) examines five identity processes: **Exploration in Breadth** relates to the degree to which a person actively explores different parental identity alternatives and assesses them according to one’s goals, values, and beliefs, before making commitments. **Commitment Making** refers to the extent to which a person has made choices about important parental identity-relevant issues, as well as accepting and executing these choices. These first two dimensions represent the first cycle of commitment formation (Luyckx et al., 2008). **Exploration in Depth** relates to the degree of deep internal investigation of the chosen parental identities (commitments) and their compatibility with a person’s standards and values. **Identification with Commitment** refers to the extent a person feels confident and in harmony with their choices (commitments) regarding parental identity issues (Eliakim et al., 2025). These two dimensions represent the second cycle of the evaluation of existing commitments (Luyckx et al., 2008). **Ruminative Exploration** refers to a less adaptive exploration that expresses dispersion and hesitation in making commitments on important parental identity-related issues (Eliakim et al., 2025).

This model offers several advantages for the present study. The five distinct dimensions enable a detailed analysis of complex identity processes with greater nuance (Luyckx et al., 2008). The model distinguishes between adaptive and maladaptive forms of exploration, capturing both positive and negative aspects of identity development (Luyckx et al., 2008)—particularly valuable for understanding adjustment to parenthood. As a dual-cycle model, it captures both commitment formation and evaluation processes (Luyckx et al., 2011). Finally, its dynamic nature (Luyckx et al., 2008, 2011) makes it well-suited for research examining parental identity development over time rather than at a single time point and, as we argue in this study, for understanding how these identity processes may predict personal growth during this major developmental transition. In addition, this multidimensional structure is particularly valuable for examining personal growth, as it allows us to identify which specific identity processes—rather than parental identity development as a whole—predict positive psychological outcomes during this major developmental transition.

#### 1.2.2. Personal Growth and the Transition to Parenthood

The concept of personal growth (PG; derived from the post-traumatic growth literature; Tedeschi & Calhoun, 1996, 2004) refers to positive psychological change that occurs as a result of effective coping with a challenging and significant life event (Tedeschi & Calhoun, 2004). According to Tedeschi and Calhoun (1996), this positive change unfolds across five domains: personal strength, appreciation of life, spirituality, new possibilities, and close relationships with others. The transition to parenthood is a normative and significant life event that entails coping with considerable demands and pressures and may therefore serve as a fertile ground for PG (Taubman-Ben-Ari et al., 2011).

Taubman-Ben-Ari and colleagues were the first to establish the connection between PG and the transition to parenthood (Taubman-Ben-Ari, 2025; Taubman-Ben-Ari et al., 2011, 2019), and over the years, their work has progressively advanced our understanding of PG in the context of becoming a parent (Taubman-Ben-Ari, 2019, 2025). Their studies have examined PG across a range of timing, contexts, and circumstances, including among mothers during pregnancy (Taubman-Ben-Ari et al., 2022), mothers across a broad range of demographic backgrounds and birth-related circumstances (Taubman-Ben-Ari, 2025), mothers and fathers at various points postpartum (Porat-Zyman et al., 2017), fathers whose infants were conceived through assisted reproductive technology (Taubman-Ben-Ari et al., 2018), and women undergoing medically assisted reproduction (Mijalevich-Soker et al., 2025).

Beyond examining the occurrence of PG across these varied contexts, this body of research has also identified key factors associated with PG, including personal resources such as optimism, positive and negative emotions, parenting stress (Taubman-Ben-Ari et al., 2018), self-mastery, emotional regulation, and social sharing (Mijalevich-Soker et al., 2025), as well as social support from family members and a significant other (Taubman-Ben-Ari, 2022).

Building on this growing body of research, the present study seeks to enrich our understanding of PG in the context of becoming a parent by introducing parental identity development as a theoretical framework that has not yet been examined in this context. While previous research has identified various personal and social factors associated with PG, the theoretical framework of parental identity development offers a unique lens—one that focuses on the internal identity processes through which individuals navigate this major life transition. Specifically, we examine how five distinctive identity development processes predict changes in PG across two critical time points during this transition.

### 1.3. The Present Study

The way individuals experience and navigate the challenging period of the transition to parenthood has widespread effects on parents, children, and families (Don et al., 2014; Pancer et al., 2000). Parental identity development is an important factor that relates strongly to parents’ well-being during this transition (Delmore-Ko et al., 2000); therefore, examining the underlying processes of parental identity development using a reliable, detailed, process-oriented identity model is important. Additionally, while research has predominantly focused on negative outcomes during this transition, there is growing recognition that it can also catalyze positive psychological change such as personal growth (Taubman-Ben-Ari et al., 2011). Given that PG has been characterized as an ongoing developmental experience whose first beneficial signs can be identified within a relatively short period (Porat-Zyman et al., 2017; Taubman-Ben-Ari et al., 2019), a short longitudinal approach examining changes during this transition may offer a meaningful window into this experience. However, research examining how parental identity processes predict personal growth during the transition to parenthood remains scarce. This study addresses this gap by examining first-time expectant parents at two time points (T1: 1–3 months before birth; T2: 3–5 months after birth), investigating the role of parental identity development processes—three exploration processes (Exploration in Breadth, Exploration in Depth, and Ruminative Exploration) and two commitment processes (Commitment Making and Identification with Commitment)—in predicting changes in personal growth during this transition. Additionally, in an exploratory manner, we examine whether gender moderates these associations.

**Hypothesis** **1.** *Based on identity development theory suggesting positive associations between commitment dimensions (Commitment Making and Identification with Commitment) and adaptive exploration dimensions (Exploration in Breadth and Exploration in Depth) with positive adjustment outcomes, we hypothesize that these four dimensions will positively predict changes in PG. Conversely, consistent with research linking Ruminative Exploration to poorer adjustment, we hypothesize that Ruminative Exploration will negatively predict changes in PG.*

Gender considerations. The transition to parenthood represents a significant life transition for both women and men (Gross & Marcussen, 2017). Contemporary social expectations increasingly emphasize gender equality in household and family responsibilities (Mickelson & Biehle, 2017), with men now expected to assume more active and involved roles in childcare and to share parenting tasks more equally with mothers (Hrybanova et al., 2019; Lewington et al., 2021). Despite these shifting expectations, research continues to document gender differences in the experience of this transition (Cao et al., 2023; Mickelson & Biehle, 2017). Notably, such differences have also been documented in the experience of PG, though findings are inconsistent. While no gender differences in PG were found one month after birth, mothers reported significantly higher levels than fathers by five months postpartum (Porat-Zyman et al., 2017), suggesting that gender differences in PG may depend on the timing of measurement. Given the inconclusive nature of these findings, gender differences in parental identity development and PG will be examined in an exploratory manner.

## 2. Materials and Methods

### 2.1. Participants

The total sample included 169 Israeli participants (women N = 97, M_age_ = 31.9, SD = 3.46; and men N = 72, M_age_ = 32.1, SD = 3.43) who were asked to complete questionnaires during pregnancy (M_week_ = 30.98, SD = 4.33) and 3–5 months after the birth of their first child. Of the participants, 88.2% were married, and 11.8% were in a committed relationship. In terms of Jewish self-reported religiosity, the following was reported: secular, 74%; traditional, 16.6%; religious, 8.3%; Orthodox and others, 1.2%. Regarding economic status (a subjective assessment of the individual’s income compared to the Israeli average monthly income), 47.9% were below average, 29.6% average, and 22.5% above average. At T2, attrition rates were 41.2% among women (N = 40) and 26.4% among men (N = 19). To examine potential attrition bias, we compared participants who completed both measurement points with those who participated only in the first wave on all T1 variables. Independent sample *t*-tests revealed no significant differences between groups on any of the baseline measures (all *p*-values > 0.10).

### 2.2. Procedure

The sample was recruited through social media posts, which invited women and men who were expecting their first child to complete a questionnaire regarding their experience. The participants were asked to complete self-report surveys at two time points: first from 1 to 3 months before the expected date of birth (T1) and then again 3 to 5 months after the actual date of birth (T2). This study was approved by the Ethics Committee of Bar-Ilan University (protocol code 087, 14 December 2021), and participation was anonymous and voluntary. To maintain the participants’ anonymity and to be able to match the surveys across the different time points, the participants were asked to provide a unique code that included their first and last initials and date of birth.

### 2.3. Measures

#### 2.3.1. Parental Identity

To assess parental identity, participants completed the Parental Identity version of the Dimensions of Identity Development Scale (DIDS-PI; Eliakim et al., 2025), an adaptation of the original DIDS (Luyckx et al., 2008) to parenting. Five identity dimensions were assessed, and each dimension included five items: (a) Exploration in Breadth (sample item: “I am considering a number of parental styles that may be good for me”; Cronbach’s α: T1 = 0.89, T2 = 0.89); (b) Commitment Making (sample item: “I have already made a decision about what I will do in my life as a parent”; Cronbach’s α: T1 = 0.90, T2 = 0.90); (c) Exploration in Depth (sample item: “I talk to other people about my future plans regarding being a parent”; Cronbach’s α: T1 = 0.81, T2 = 0.79); (d) Identification with Commitment (sample item: “I’m sure my future as a parent is right for me”; Cronbach’s α: T1 = 0.90, T2 = 0.90); (e) Ruminative Exploration (sample item: “It is hard for me to stop thinking about the parental style I want to adopt in my life”; Cronbach’s α: T1 = 0.78, T2 = 0.85). To examine the construct validity of the DIDS-PI, an EFA was conducted using Principal Component Analysis with Promax rotation, an oblique method permitting correlated factors consistent with the theoretical model. The inspection of the scree plot supported a five-factor solution (eigenvalues = 9.98, 3.23, 1.44, 1.25, 1.06), accounting for 67.86% of the total variance. The pattern matrix showed a clear structure with items loading on their intended dimensions and minimal cross-loadings. These findings are consistent with the validation study of the DIDS-PI (Eliakim et al., 2025), which demonstrated good CFA fit (CFI = 0.92, RMSEA = 0.06) and metric measurement invariance across cohorts and genders.

#### 2.3.2. Personal Growth

Personal growth following the transition to parenthood was assessed using the Post-Traumatic Growth Inventory (PTGI), originally developed by Tedeschi and Calhoun (1996) and later adapted for the context of parenthood by Taubman-Ben-Ari et al. (2011). The questionnaire contains 21 items scored on a 6-point Likert-type rating scale, ranging from 1 (I did not experience this change) to 6 (I experienced this change to a very great degree). The items refer to the changes a person is experiencing resulting from the transition to parenthood. For each statement, participants were asked to indicate the degree to which the change occurred in their life—at T1, since pregnancy, and at T2, since birth. These items include five domains: (a) priorities, (b) relations with others, (c) significance of physical strength, (d) personal and spiritual development, and (e) life appraisal. Following previous studies that suggest consolidating these domains into a single-factor scale (Taubman-Ben-Ari & Spielman, 2014), we regarded PG as an overall variable (Cronbach’s α: T1 = 0.93, T2 = 0.92). To examine the dimensionality of the PG scale, an EFA was conducted on all 21 items using Principal Component Analysis. Both the scree plot and parallel analysis supported a single-factor solution (eigenvalue = 8.83, accounting for 42.04% of the total variance). All items loaded positively on the single factor (loadings ranged from 0.42 to 0.76), supporting the use of a composite score. It should be noted that this proportion of variance is modest for a 21-item scale spanning multiple domains; however, the high internal consistency (T1 α = 0.93, T2 α = 0.92) and consistently positive item loadings support the reliability of the composite.

#### 2.3.3. Demographic Background

This questionnaire asked the participants to report several demographic characteristics, including age, marital status, level of religiosity (secular, traditional, religious, orthodox, and other), and economic status (a subjective assessment of the individual’s income compared to the Israeli average monthly income on a scale from 1 = below, 2 = average, and 3 = above).

### 2.4. Analytical Strategy

Power analyses using G*Power v 3.1.9.7 were conducted for the different analyses included in this study, which comprised independent sample *t*-tests, repeated-measures ANOVAs, hierarchical regressions, and moderation analyses. Among these, the hierarchical regression testing whether the five identity dimensions add to the prediction of PG beyond baseline growth and age (R^2^ increase) required the largest sample size. This analysis indicated that a sample of 92 participants would be required to detect medium effects (f^2^ = 0.15) with α = 0.05 and power = 0.80. In the current study, these analyses were conducted separately for men (n = 72) and women (n = 97). This provided sufficient power for detecting medium effects among women, while power for detecting medium effects among men was somewhat limited. Post hoc power analyses (GPower v 3.1.9.7) indicated that the achieved power for the primary regression analyses was 0.79 for men and 0.91 for women.

Missing data analysis indicated 34.9% missing observations, and Little’s MCAR test (Little, 1988) for all theoretical and demographic variables yielded a non-significant result (χ^2^(46) = 40.203, *p* = 0.713), indicating that a missing data pattern was completely at random (MCAR). Therefore, missing data were imputed using the Expectation–Maximization (EM) algorithm, implemented via SPSS version 28.

The analyses proceeded in three steps. **Preliminary analyses:** First, we computed descriptive statistics and Pearson’s correlations among the five identity dimensions, PG, and age, separately for men and women. Next, we examined gender differences in each identity dimension and in PG using independent sample *t*-tests, conducted separately for T1 and T2. To assess changes over time, we conducted a series of repeated-measures ANOVAs, with time (T1 vs. T2) as a within-subjects factor and gender as a between-subjects factor. When significant time × gender interactions emerged, we conducted Bonferroni-adjusted pairwise comparisons within each gender to explore the nature of the interaction.

**Primary analyses:** To address our main research question—whether parental identity dimensions at T1 predict changes in PG—we conducted hierarchical regression analyses separately for men and women. In each model, T1 PG was entered in the first step, followed by age in the second step and the five identity dimensions (entered using the Stepwise method) in the third step. This allowed us to assess the unique contribution of identity dimensions to PG at T2, above and beyond baseline growth and age.

**Exploratory analyses**: Based on the observed changes in identity and growth, and to further explore potential gender-based mechanisms, we conducted a set of exploratory moderation analyses using the PROCESS macro (version 4.0; Hayes, 2022). For each identity dimension, a change score (Δ) was computed by subtracting T1 from T2. We then tested whether gender moderated the association between each identity change score and T2 PG, controlling for baseline growth. These analyses were exploratory in nature and aimed to test whether gender moderates the relationship between identity development and personal growth. Given the increased number of statistical tests conducted across all analytical levels, the possibility of Type I errors should be acknowledged; the findings from the exploratory analyses in particular should be interpreted with appropriate caution and replicated in future research.

## 3. Results

### 3.1. Descriptive Statistics

Table 1 presents the means, standard deviations, and Pearson’s correlations between the five identity dimensions and PG across time points, separately for women (above the diagonal) and men (below the diagonal).

Among women, significant positive correlations were consistently found among Exploration in Breadth, Commitment Making, Exploration in Depth, and Identification with Commitment at both T1 and T2. Ruminative Exploration was positively related to Exploration in Breadth and Exploration in Depth at both time points and to Commitment Making and Identification with Commitment only at T1. Personal growth at T1 was positively associated with Exploration in Depth and Identification with Commitment and showed weaker associations with Exploration in Breadth and Ruminative Exploration. At T2, PG was correlated with Exploration in Breadth, Exploration in Depth, and Identification with Commitment and marginally with Commitment Making and Ruminative Exploration. Age was negatively related to Exploration in Breadth at T1 and to Ruminative Exploration and (marginally) to PG at T2.

Among men, Exploration in Breadth, Commitment Making, Exploration in Depth, and Identification with Commitment were positively intercorrelated at both time points. Ruminative Exploration was related to Exploration in Breadth and Exploration in Depth at both T1 and T2. At T1, PG was weakly associated with Identification with Commitment, but at T2, it was not significantly related to any of the identity dimensions. Age was not significantly correlated with any of the study variables.

### 3.2. Preliminary Analysis

#### 3.2.1. Gender Differences

Independent sample *t*-tests were conducted to examine gender differences in identity dimensions and PG at T1 and T2. Women reported significantly higher levels of Exploration in Depth at both time points. A marginally significant difference was also found in Identification with Commitment at T1, with women reporting higher levels than men. In contrast, men reported significantly higher PG at T1, while women reported significantly higher PG at T2. No significant gender differences were found in Commitment Making, Exploration in Breadth, or Ruminative Exploration at either time point. The results are presented in Table 2.

#### 3.2.2. Changes over Time

**Figure 1:**
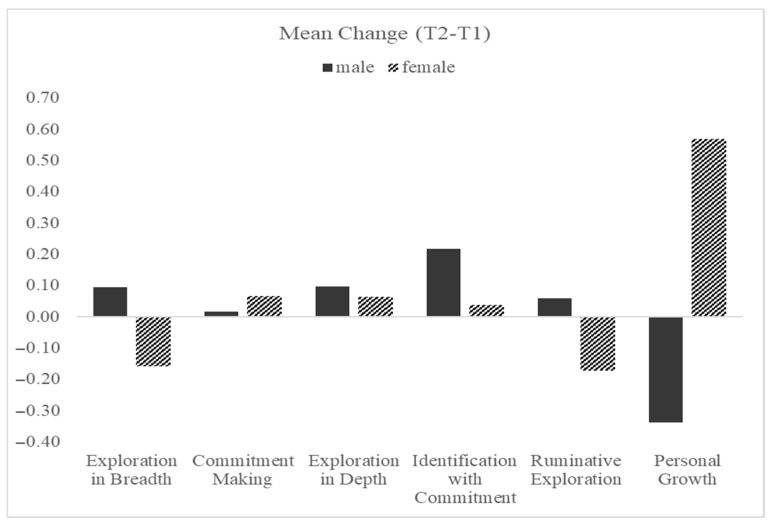
Mean change scores (T2 − T1; Y-axis) in identity dimensions and personal growth (X-axis), separately for men and women.

To examine changes over time in identity dimensions and PG, we conducted a series of repeated-measures ANOVAs with time (T1 vs. T2) as a within-subjects factor and gender (0 = men, 1 = women) as a between-subjects factor. Separate analyses were conducted for each identity dimension—Exploration in Breadth, Commitment Making, Exploration in Depth, Identification with Commitment, and Ruminative Exploration—as well as for PG. These analyses allowed us to test not only for overall changes in each construct over time but also whether these changes differed by gender. A summary of the mean change scores (T2 − T1) by gender across the different dimensions is presented in Figure 1, illustrating both the direction and magnitude of change in each group.

Exploration in Breadth. There was no significant main effect of time, *F*(1, 167) = 0.24, *p* = 0.628, indicating no overall change in Exploration in Breadth between time points. However, the time X gender interaction approached significance, *F*(1, 167) = 3.60, *p* = 0.060, suggesting a possible gender difference in change patterns. To further explore the time X gender interaction, we conducted Bonferroni-adjusted pairwise comparisons within each gender group. Among men, Exploration in Breadth remained relatively stable between T1 (M = 3.20, SE = 0.11) and T2 (M = 3.29, SE = 0.10), with no significant change over time (*p* = 0.353). Among women, however, Exploration in Breadth decreased from T1 (M = 3.34, SE = 0.09) to T2 (M = 3.18, SE = 0.08), with this decline approaching statistical significance (*p* = 0.070).

Commitment Making. The results revealed no significant main effect of time, *F*(1, 167) = 0.37, *p* = 0.544, nor significant time X gender interaction, *F*(1, 167) = 0.14, *p* = 0.710, indicating that the levels of Commitment Making remained stable over time and did not differ between men and women in their trajectories. Pairwise comparisons adjusted using Bonferroni correction showed no significant within-group changes. Among men, Commitment Making scores remained nearly unchanged from T1 (*M* = 3.13, *SE* = 0.10) to T2 (*M* = 3.14, *SE* = 0.10), *p* = 0.876. Similarly, women showed no significant change, with scores changing from T1 (*M* = 3.12, *SE* = 0.09) to T2 (*M* = 3.18, *SE* = 0.09), *p* = 0.454.

Exploration in Depth. The results revealed no significant main effect of time, *F*(1, 167) = 2.04, *p* = 0.155, and no significant time X gender interaction, *F*(1, 167) = 0.09, *p* = 0.761, suggesting that changes in Exploration in Depth over time were similar for men and women. Bonferroni-adjusted pairwise comparisons further indicated that neither men nor women exhibited significant within-group changes. Among men, Exploration in Depth did not change significantly from T1 (*M* = 3.01, *SE* = 0.10) to T2 (*M* = 3.11, *SE* = 0.09), *p* = 0.255. Among women, scores also did not change significantly from T1 (*M* = 3.36, *SE* = 0.09) to T2 (*M* = 3.42, *SE* = 0.08), *p* = 0.391.

Identification with Commitment. The results revealed a significant main effect of time, *F*(1, 167) = 4.48, *p* = 0.036, η^2^ = 0.026, indicating an overall increase in Identification with Commitment from T1 to T2. The time X gender interaction was not significant, *F*(1, 167) = 2.21, *p* = 0.139, suggesting that the pattern of change did not differ significantly by gender. Bonferroni-adjusted pairwise comparisons revealed that men showed a significant increase in Identification with Commitment from T1 (*M* = 3.18, *SE* = 0.11) to T2 (*M* = 3.39, *SE* = 0.10), *p* = 0.019. For women, the increase from T1 (*M* = 3.43, *SE* = 0.09) to T2 (*M* = 3.47, *SE* = 0.08) was not significant, *p* = 0.630.

Ruminative Exploration. There was no significant main effect of time, *F*(1, 167) = 0.75, *p* = 0.388, but the time X gender interaction approached significance, *F*(1, 167) = 3.19, *p* = 0.076, suggesting a potential difference in the pattern of change between men and women. To further explore this interaction, we conducted Bonferroni-adjusted pairwise comparisons within each gender group. Among men, Ruminative Exploration did not change significantly from T1 (M = 2.44, SE = 0.10) to T2 (M = 2.50, SE = 0.10), *p* = 0.545. Among women, Ruminative Exploration significantly decreased from T1 (M = 2.50, SE = 0.09) to T2 (M = 2.33, SE = 0.09), *p* = 0.044.

Personal Growth. The results revealed a marginal main effect of time, *F*(1, 167) = 3.41, *p* = 0.067, and a significant time X gender interaction, *F*(1, 167) = 52.81, *p* < 0.001, indicating that changes in PG over time differed by gender. To further explore the interaction, we conducted Bonferroni-adjusted pairwise comparisons within each gender. Among men, PG decreased significantly from T1 (M = 3.98, SE = 0.11) to T2 (M = 3.64, SE = 0.11), *p* < 0.001. In contrast, women showed a significant increase in PG from T1 (M = 3.61, SE = 0.09) to T2 (M = 4.18, SE = 0.09), *p* < 0.001.

### 3.3. Primary Analyses: Parental Identity Dimensions as Predictors of Personal Growth

Hierarchical regression analyses were conducted separately for men and women to examine whether parental identity dimensions at T1 predicted PG at T2, above and beyond baseline PG and age. The results are presented in Table 3.

For both men and women, baseline PG (Step 1) was a strong significant predictor of T2 PG (men: β = 0.51, *p* < 0.001, R^2^ = 0.259; women: β = 0.68, *p* < 0.001, R^2^ = 0.467). Age contributed significantly for women only (β = −0.16, *p* = 0.031, ΔR^2^ = 0.026) but not for men (β = −0.12, *p* = 0.237). In Step 3, the addition of parental identity dimensions explained a significant and equivalent increment in variance for both groups (ΔR^2^ = 0.071, *p* = 0.009 for men; ΔR^2^ = 0.071, *p* < 0.001 for women). In both cases, Identification with Commitment was the sole significant predictor retained by the Stepwise procedure, positively predicting PG at T2 (men: β = 0.28, *p* = 0.009; women: β = 0.29, *p* < 0.001), with all prior effects remaining robust.

### 3.4. Exploratory Analyses: Moderation and Change Score Analyses

To further explore potential gender-based mechanisms, we examined whether gender moderated the relationship between changes in parental identity dimensions and PG at T2. For each identity dimension, a change score (Δ) was computed by subtracting T1 from T2. The means, standard deviations, ranges, and Pearson’s correlations of the change scores are presented in Table 4.

Five moderation analyses were then conducted using the SPSS PROCESS macro (v4.0; Hayes, 2022), with gender as the moderator, each identity change score as the predictor, and PG at T1 as a covariate. The results are summarized in Table 5.

**Figure 2:**
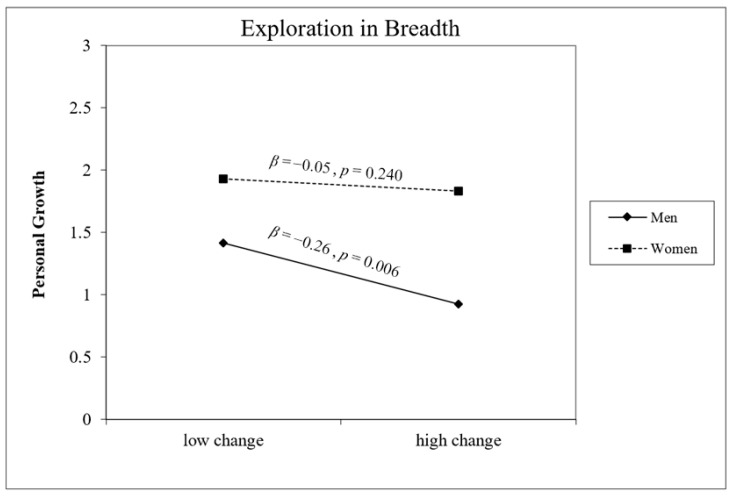
Gender as a moderator of the association between change in Exploration in Breadth and personal growth at T2 (controlling for baseline PG). Conditional effects are shown for men (solid line, Β = −0.26, *p* = 0.006) and women (dashed line, Β = −0.05, *p* = 0.240).

**Figure 3:**
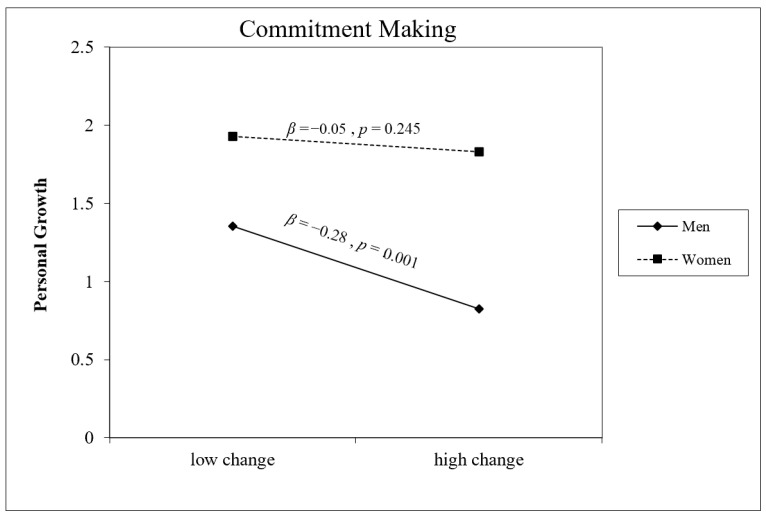
Gender as a moderator of the association between change in Commitment Making and personal growth at T2 (controlling for baseline PG). Conditional effects are shown for men (solid line, Β = −0.28, *p* = 0.001) and women (dashed line, Β = −0.05, *p* = 0.245).

**Figure 4:**
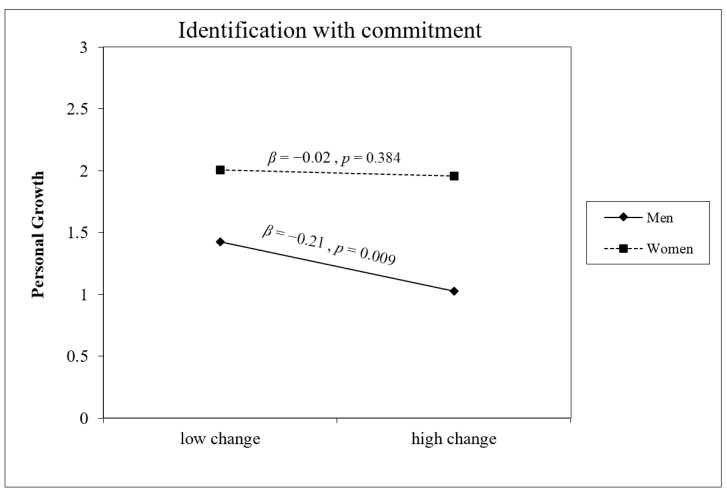
Gender as a moderator of the association between change in Identification with Commitment and personal growth at T2 (controlling for baseline PG). Conditional effects are shown for men (solid line, Β = −0.21, *p* = 0.009) and women (dashed line, Β = −0.02, *p* = 0.384).

Gender was a significant main effect across all five models, with women consistently reporting higher PG at T2 than men. Significant gender × change interactions emerged for Exploration in Breadth (B = 0.23, *p* = 0.047) and Commitment Making (B = 0.24, *p* = 0.028), and a marginally significant interaction was found for Identification with Commitment (B = 0.23, *p* = 0.059). As illustrated in Figure 2, Figure 3 and Figure 4, for all three dimensions, the association between identity change and PG was negative and significant for men (Exploration in Breadth: B = −0.29, *p* = 0.006; Commitment Making: B = −0.30, *p* = 0.001; Identification with Commitment: B = −0.26, *p* = 0.009) but non-significant for women (all *p*s > 0.24). No significant interactions were found for Exploration in Depth (B = 0.20, *p* = 0.095) or Ruminative Exploration (B = −0.04, *p* = 0.378).

## 4. Discussion

This study examined parental identity development processes as predictors of personal growth during the critical period of the transition to parenthood, revealing a complex pattern of findings that varied across identity dimensions and gender. While the design focused on the predictive role of parental identity processes, the findings offer an opportunity to consider how these two theoretical frameworks may illuminate one another—with parental identity development shedding light on the conditions that enable personal growth and personal growth outcomes in turn revealing the complexity and nuance of parental identity development during this pivotal period. The discussion addresses these findings by examining the predictive role of Identification with Commitment, the changing correlation patterns among identity dimensions over time and their possible role in explaining why adaptive exploration dimensions did not predict PG. It also considers gender differences in both personal growth trajectories and patterns of parental identity development.

### 4.1. Parental Identity Development Processes as Predictors of PG

#### 4.1.1. The Central Role of Identification with Commitment

Our primary research question examined whether parental identity development dimensions measured during pregnancy predict changes in PG from the prenatal period to early postpartum. Although we hypothesized that both commitment dimensions (Commitment Making and Identification with Commitment) and the adaptive exploration dimensions (Exploration in Breadth and Exploration in Depth) would predict positive changes in PG, only Identification with Commitment emerged as a significant predictor for both men and women. This finding aligns with the literature that distinguishes between these two commitment processes: while Commitment Making indicates that identity decisions have been made (Luyckx et al., 2006), Identification with Commitment reflects a deeper integration of commitments with the self, evoking feelings of confidence and certainty (Luyckx et al., 2008, 2013). From an identity development perspective, this finding demonstrates that such deep integration of the parental role into one’s core identity can already occur during pregnancy—before the role becomes a lived reality—and that it is associated with distinctive psychological outcomes and adjustment (Luyckx et al., 2013; Mannerström et al., 2017). From a personal growth perspective, these findings may tentatively suggest that personal growth following a major life transition does not emerge solely from the experience of challenge itself. Rather, it may require a degree of internal readiness—a sense of harmony and confidence with one’s anticipated parental role—that is established even before the transition fully unfolds.

#### 4.1.2. Correlations Shift Between Identity Processes: Implications for Predicting PG

The changing correlation patterns among the five identity dimensions across time points, as reflected in Table 1, may reflect the dynamic nature of parental identity development during this critical period, thereby helping explain why adaptive exploration dimensions did not predict PG. At T1 (prenatal), moderate correlations emerged between adaptive exploration dimensions and Ruminative Exploration among both genders. Additionally, significant moderate correlations were found between commitment dimensions and Ruminative Exploration among women. At T2 (postnatal), this pattern shifted: correlations between adaptive exploration dimensions and Ruminative Exploration remained stable across both genders, while correlations between commitment dimensions and Ruminative Exploration became non-significant. These findings suggest that before birth, when the parental role is not yet embodied in reality, Ruminative Exploration (the maladaptive exploration dimension) connects to commitment processes as expectant parents grapple with anticipatory identity formation. However, once parenthood becomes embodied in its earliest stages, commitment processes appear to stabilize in response to immediate practical demands and role enactment. In contrast, exploration processes require more time to stabilize and develop associations with positive psychological outcomes (PG). This may explain why correlations between adaptive exploration dimensions and Ruminative Exploration remained stable across the two time points, while commitment-related patterns shifted.

### 4.2. Gender Differences During the Transition to Parenthood

#### 4.2.1. Gender Differences in Personal Growth

Significant gender differences emerged in PG during the transition to parenthood. Women showed an increase in PG from T1 to T2, while men showed a decrease—a finding that, while consistent with our hypothesis regarding higher female growth, was unexpected regarding male decline. Despite efforts toward gender equality in parenting (Mickelson & Biehle, 2017), mothers still experience greater life changes during early parenthood (Cao et al., 2023; Ruppanner et al., 2019), resulting in more central and intense parental role perception that is associated with PG (Taubman-Ben-Ari et al., 2012). This centrality may be rooted in early socialization, where girls are often steered toward nurturing and family-oriented roles, such that by middle childhood they already tend to expect significantly higher levels of family orientation than boys (Endendijk & Portengen, 2022). The early preparation facilitates a deeper perception of the caregiving role, reflecting women’s self-concept that is more intertwined with motherhood than men’s self-concept with fatherhood (Kaźmierczak & Karasiewicz, 2019).

In contrast, new fathers often function as secondary caregivers who receive less recognition for their transition experiences (Brunstad et al., 2020; Machin, 2015; Škvařil & Presslerová, 2024). This marginalized position may lead them to push aside their needs and feelings (Brunstad et al., 2020), making them less likely to recognize or be prepared to experience inner psychological changes such as PG. While this interpretation is plausible, the unexpected nature of this finding warrants further investigation.

These findings suggest that PG follows role centrality—those with more recognized and central parenting roles experience greater PG. Therefore, promoting fathers’ early involvement and recognition could support their experience of positive psychological outcomes during the transition to parenthood, rather than leaving them to wait for their role to naturally expand over time (Doherty et al., 2006; Riina & Feinberg, 2012). The unexpected decline in PG among men, and its counterintuitive association with increased identity work, is further examined in the following section.

#### 4.2.2. Gender Differences in Parental Identity Development

Gender differences also emerged in parental identity development during the transition to parenthood. Exploratory analyses revealed that only changes in Exploration in Breadth, Commitment Making, and Identification with Commitment predicted changes in PG, and this was found exclusively among men. Notably, and counterintuitively, higher levels of these identity processes predicted a decrease rather than an increase in PG, suggesting that identity work itself may carry a psychological cost during this critical period. To explain this finding, we must consider men’s distinct experience in developing parental identity during this critical period. Unlike women, men do not undergo biological changes that may prime thoughts about the transition to parenthood during pregnancy; therefore, they may postpone paternal identity development until after their child’s birth (Bataille & McGil-Carlison, 2017). This lack of biological priming is further compounded by childhood socialization experiences that shape lower family orientation expectations in boys (Endendijk & Portengen, 2022). Consequently, as men enter parenthood less prepared by their prior social experiences than women (Barrret & Charlton, 2025; Kaźmierczak & Karasiewicz, 2019), their paternal identity work prior to birth remains more abstract or conceptual (Solberg et al., 2023). From a role theory perspective, this reflects how culturally defined role expectations influence the way individuals internalize and enact specific rules (Turner, 2001), with men’s paternal role remaining less socially defined and recognized (Kuscul & Adamsons, 2022; Lewington et al., 2021). The lack of embodied experience may limit the depth of their identity work. Furthermore, interactional approaches, such as those suggested by Stryker’s identity theory (Serpe & Stryker, 2011), might add that men have fewer social interactions surrounding fatherhood. This may help explain why the commitment formation cycle may require more time for new fathers.

The findings suggest that high levels of Identification with Commitment during pregnancy may represent a conceptual integration of the parental role that is not yet grounded in lived reality. When confronted with the reality of fatherhood, these commitments may require renegotiation: while fathers are making commitments and identifying with their new parental role, they are simultaneously still exploring different paternal identity possibilities, leading to renewed exploration that is associated with decreased PG. It is possible that this pattern reflects a form of developmental regression, though this interpretation remains speculative and requires further investigation. This aligns with research indicating that men require time to develop their paternal role—a process that typically unfolds during pregnancy and continues for approximately one year after birth (Solberg et al., 2023). Taken together, these findings tentatively suggest that authentic personal growth requires not only conceptual identity formation but identity that is grounded in lived experience and social interactions—a process that, for new fathers, may unfold on a longer developmental timeline that captured in the present study.

Fathers today face increasingly complex identity construction tasks. Given evolving social expectations that fathers should be more present and take an integral part in childcare, alongside their traditional role as primary breadwinners, there is now a broader range of possible paternal models available. This may make the task of constructing paternal identity more complex than in the past (Bataille & McGil-Carlison, 2017; Bosoni & Mazzucchelli, 2019). Furthermore, new fathers must also navigate the tension between longstanding masculine ideals and shifting cultural expectations of manhood (Segev, 2025). This complexity may further compound the challenges fathers experience during this critical period.

These findings regarding gender differences in both PG trajectories and parental identity development patterns raise a critical theoretical question: do men and women follow parallel developmental pathways—in both PG and parental identity formation—at different rates, with fathers experiencing delayed progression due to their distinct experience of the parental transition, or do they traverse fundamentally different developmental trajectories? Determining whether these patterns reflect differences in developmental timing or qualitatively distinct pathways requires extended longitudinal research that tracks both identity development and PG well beyond the early postpartum period. Additionally, these gender differences raise a methodological question: should longitudinal parental identity research examine both genders together or investigate them separately?

### 4.3. Limitations

This study has several limitations. The correlational and predictive nature of this study precludes causal inferences. While our longitudinal design allows us to examine whether parental identity dimensions predict changes in PG over time, we cannot determine whether these identity processes cause changes in PG or whether other unmeasured variables account for these associations. Furthermore, the longitudinal findings may be susceptible to threats to internal validity, such as history effects (e.g., differential parental leave durations or unforeseen life events) and maturity effects linked to distinct gender socialization experiences (Endendijk & Portengen, 2022; Kaźmierczak & Karasiewicz, 2019; Lewington et al., 2021). These could represent alternative explanations for the observed developmental trajectories. Additionally, the short time frame provides only an initial view of these processes. Longer longitudinal studies, extending at least one-year post-birth, are essential for understanding parental identity development in general and paternal identity development in particular and would help determine whether the gender differences observed reflect different developmental timelines or fundamentally distinct trajectories. Finally, the relatively small sample limits generalizability, and larger samples are needed to capture the full variability in parental identity development (Piotrowski, 2021a) and to enable more detailed analyses, such as examining potential moderators of the relationships between parental identity processes and PG.

### 4.4. Implications

Theoretical and Clinical: This study contributes to a broader theoretical understanding by examining parental identity development and personal growth as mutually enriching frameworks. The findings demonstrate that parental identity processes—particularly Identification with Commitment—contribute to our understanding of personal growth by identifying specific internal mechanisms that predict its emergence during the transition to parenthood. At the same time, personal growth outcomes enrich our understanding of parental identity development by revealing the complexity and nuance of identity formation during this period, particularly among fathers. Together, these frameworks offer a more complete picture of the psychological experience of becoming a parent than either framework could provide alone.

The findings also underscore the value of gender-differentiated approaches when studying parental identity development. The complex pattern observed among fathers—where increases in identity dimensions were associated with decreases in personal growth—suggests that traditional models of identity development may be insufficient for capturing the distinct experience of paternal identity formation during this critical transition.

From a clinical perspective, healthcare providers have an opportunity to expand beyond technical childcare preparation to address the deeper psychological processes of role identification. Interventions should encourage men to begin identity exploration work earlier in pregnancy, and support should extend well beyond the immediate postpartum period. Expectant fathers should be prepared that commitments formed during pregnancy may require renegotiation after birth and that this is a normal part of authentic development.

## 5. Conclusions

The present study yielded a complex pattern of findings that advance our understanding of the role of parental identity development in predicting personal growth during the transition to parenthood. Identification with Commitment emerged as the sole predictor of personal growth for both men and women, while changes in identity dimensions predicted changes in personal growth exclusively among men. These gender differences suggest that mothers and fathers may follow distinct developmental pathways during this transition, though extended longitudinal research is needed to determine whether these patterns reflect differences in timing or fundamentally distinct trajectories. By bringing together the frameworks of parental identity development and personal growth, this study suggests that these two perspectives are mutually informative: parental identity processes predict the emergence of personal growth, while personal growth outcomes in turn reveal the authenticity and depth of parental identity formation during this pivotal period.

## Figures and Tables

**Table 1:** Correlations, means, and standard deviations for identity dimensions and personal growth at T1 and T2, by gender.

			1	2	3	4	5	6	7	8	9	10	11	12	13	M	SD
T1	1	Exploration in Breadth		0.51 ***	0.66 ***	0.60 ***	0.50 ***	0.23 *	0.45 ***	0.30 **	0.50 ***	0.45 ***	0.26 *	0.41 ***	−0.13	3.34	0.91
	2	Commitment Making	0.44 ***		0.56 ***	0.73 ***	0.30 **	0.19 ^†^	00.20 ^†^	0.54 ***	0.37 ***	0.61 ***	0.14	0.35 ***	−0.04	3.12	0.94
	3	Exploration in Depth	0.66 ***	0.49 ***		0.65 ***	0.44 ***	0.40 ***	0.37 ***	0.47 ***	0.61 ***	0.48 ***	0.31 **	0.44 ***	−0.04	3.36	0.82
	4	Identification with Com	0.33 **	0.66 ***	0.46 ***		0.28 **	0.36 **	0.20 *	0.36 ***	0.35 ***	0.69 ***	0.07	0.51 ***	−0.10	3.43	0.98
	5	Ruminative Exploration	0.52 ***	0.02	0.59 ***	0.03		0.23 *	0.33 **	0.06	0.37 ***	0.03	0.49 ***	0.29 **	−0.25 *	2.50	0.91
	6	Personal Growth	−0.03	0.08	0.15	0.24 *	0.04		0.24 *	0.13	0.31 **	0.26 *	0.21 *	0.68 ***	−0.09	3.61	0.99
T2	7	Exploration in Breadth	0.59 ***	0.14	0.45 ***	0.03	0.48 ***	0.12		0.27 **	0.59 ***	0.27 **	0.53 ***	0.38 ***	−0.06	3.18	0.83
	8	Commitment Making	0.12	0.43 ***	0.19	0.21 ^†^	−0.03	0.23 *	0.27 *		0.53 ***	0.69 ***	0.10	0.26 **	−0.08	3.18	0.92
	9	Exploration in Depth	0.55 ***	0.33 **	0.57 ***	0.29 *	0.53 ***	0.13	0.66 ***	0.33 **		0.42 ***	0.41 ***	0.46 ***	−0.16	3.42	0.76
	10	Identification with Com	0.06	0.41 ***	0.09	0.39 **	−0.18	0.12	0.11	0.53 ***	0.12		0.03	0.43 ***	−0.02	3.47	0.95
	11	Ruminative Exploration	0.28 *	−0.01	0.32 **	0.08	0.58 ***	0.07	0.50 ***	−0.02	0.66 ***	−0.03		0.25 *	−0.23 *	2.32	0.87
	12	Personal Growth	0.23 ^†^	0.28 *	0.20	0.39 **	−0.03	0.51 ***	0.11	0.05	0.12	0.12	0.00		−0.22 *	4.18	0.93
	13	Age	−0.03	−0.23 ^†^	−0.06	−0.10	−0.05	−0.01	0.08	−0.11	−0.12	−0.02	−0.01	−0.13		31.94	3.45
	M		3.20	3.13	3.01	3.18	2.44	3.98	3.29	3.14	3.11	3.39	2.50	3.64	32.19		
	SD		0.90	0.80	0.86	0.81	0.76	0.80	0.80	0.83	0.77	0.61	0.84	0.91	3.43		

**Note.** Correlations for men are presented below the diagonal and correlations for women above the diagonal. T1 = Time 1; T2 = Time 2. * *p* < 0.05, ** *p* < 0.01, *** *p* < 0.001, ^†^ *p* < 0.08.

**Table 2:** Means and standard deviations for identity dimensions and personal growth by gender (T1 and T2).

		Men		Women		t(df)	*p*-Value
		M	SD	M	SD		
T1	Exploration in Breadth	3.2	0.9	3.34	0.91	−1.01 (167)	0.314
	Commitment Making	3.13	0.8	3.12	0.94	0.07 (167)	0.945
	Exploration in Depth	3.01	0.86	3.36	0.82	−2.63 (167)	0.009
	Identification with Com	3.18	0.81	3.43	0.98	−1.85 (165.13 ^a^)	0.067
	Ruminative Exploration	2.44	0.76	2.5	0.91	−0.40 (167)	0.693
	Personal Growth	3.98	0.8	3.61	0.99	2.60 (167)	0.010
T2	Exploration in Breadth	3.29	0.8	3.18	0.83	0.87 (167)	0.386
	Commitment Making	3.14	0.83	3.18	0.92	−0.30 (167)	0.762
	Exploration in Depth	3.11	0.77	3.42	0.76	−2.59 (167)	0.010
	Identification with Com	3.39	0.61	3.47	0.95	−0.62 (164.27 ^a^)	0.533
	Ruminative Exploration	2.5	0.84	2.32	0.87	1.35 (167)	0.180
	Personal Growth	3.64	0.91	4.18	0.93	−3.76 (167)	0.000

^a^ Welch’s correction applied due to unequal variances (Levene’s test), resulting in non-integer degrees of freedom.

**Table 3:** Hierarchical regression analyses predicting personal growth at T2, separately for men and women.

	Men (*n* = 72)			Women (*n* = 97)		
Predictor	*β*	R^2^	ΔR^2^	*Β*	R^2^	ΔR^2^
Step 1		0.259 ***	0.259 ***		0.467 ***	0.467 ***
PG T1	0.51 ***			0.68 ***		
Step 2		0.274 ^†^	0.015		0.493 ***	0.026 *
PG T1	0.53 ***			0.71 ***		
Age	−0.12			−0.16 *		
Step 3		0.345 **	0.071 **		0.564 ***	0.071 ***
PG T1	0.50 ***			0.68 ***		
Age	−0.13			−0.17 **		
Identification with Com.	0.28 **			0.29 ***		

Note. Only the identity dimension retained by the Stepwise procedure is shown in Step 3. PG = Personal growth. ^†^ *p* < 0.08. * *p* < 0.05. ** *p* < 0.01. *** *p* < 0.001.

**Table 4:** Correlations, means, and standard deviations for changes in identity dimensions, by gender.

		1	2	3	4	5	Min	Max	M	SD
1	Change in Exploration in Breadth		0.29 **	0.43 ***	0.27 **	0.42 ***	−2.6	1.83	−0.16	0.92
2	Change in Commitment Making	0.47 ***		0.30 **	0.59 ***	0.22 *	−2.4	2.4	0.07	0.89
3	Change in Exploration in Depth	0.40 **	0.31 **		0.35 ***	0.19	−2	1.94	0.06	0.7
4	Change in Identification with Com	0.40 **	0.50 ***	0.25 *		0.27 **	−2.2	2.4	0.04	0.75
5	Change in Ruminative Exploration	0.32 **	0.03	0.50 ***	0.05		−2.2	2.62	−0.17	0.9
	Min	−2.2	−2.2	−2	−2.18	−1.8				
	Max	3.2	2.2	2.8	2.4	2.6				
	M	0.09	0.02	0.1	0.22	0.06				
	SD	0.77	0.87	0.76	0.8	0.74				

Note. Correlations for men are presented below the diagonal and correlations for women above the diagonal. * *p* < 0.05, ** *p* < 0.01, *** *p* < 0.001.

**Table 5:** Moderation analyses: gender as moderator of identity change scores on personal growth at T2 (controlling for PG at T1).

Identity Dimension (Δ)	Main Effect of Δ	Main Effect of Gender	Interaction (Δ × Gender)	Model R^2^
	*B* (*p*)	*B* (*p*)	*B* (*p*)	
Exploration in Breadth	−0.15 (0.010) *	0.73 (<0.001) ***	0.23 (0.047) *	0.452
Commitment Making	−0.16 (0.006) **	0.78 (<0.001) ***	0.24 (0.028) *	0.461
Exploration in Depth	0.01 (0.439)	0.77 (<0.001) ***	0.20 (0.095)	0.434
Identification with Commitment	−0.13 (0.043) *	0.73 (<0.001) ***	0.23 (0.059) ^†^	0.448
Ruminative Exploration	−0.01 (0.435)	0.77 (<0.001) ***	−0.04 (0.378)	0.429

Note. Δ = change score (T2 − T1). Gender-coded 0 = men, 1 = women. All models control for PG at T1. ^†^ *p* < 0.08. * *p* < 0.05. ** *p* < 0.01. *** *p* < 0.001.

## Data Availability

The data presented in this study are available on request from the corresponding author. The data are not publicly available due to privacy and ethical restrictions.
